# 3D bi-directional transformer U-Net for medical image segmentation

**DOI:** 10.3389/fdata.2022.1080715

**Published:** 2023-01-06

**Authors:** Xiyao Fu, Zhexian Sun, Haoteng Tang, Eric M. Zou, Heng Huang, Yong Wang, Liang Zhan

**Affiliations:** ^1^Department of Electrical and Computer Engineering, University of Pittsburgh, Pittsburgh, PA, United States; ^2^Department of Biomedical Engineering, Washington University in St. Louis, St. Louis, MO, United States; ^3^Montgomery Blair High School Maryland, 51 University Blvd E, Silver Spring, MD, United States; ^4^Department of Electrical and Systems Engineering, Washington University in St. Louis, St. Louis, MO, United States; ^5^Department of Obstetrics and Gynecology, Washington University in St. Louis, St. Louis, MO, United States; ^6^Department of Radiology, Washington University in St. Louis, St. Louis, MO, United States

**Keywords:** semantic segmentation, COVID, lung, placenta, transformer, 3D UNet, CT, MRI

## Abstract

As one of the popular deep learning methods, deep convolutional neural networks (DCNNs) have been widely adopted in segmentation tasks and have received positive feedback. However, in segmentation tasks, DCNN-based frameworks are known for their incompetence in dealing with global relations within imaging features. Although several techniques have been proposed to enhance the global reasoning of DCNN, these models are either not able to gain satisfying performances compared with traditional fully-convolutional structures or not capable of utilizing the basic advantages of CNN-based networks (namely the ability of local reasoning). In this study, compared with current attempts to combine FCNs and global reasoning methods, we fully extracted the ability of self-attention by designing a novel attention mechanism for 3D computation and proposed a new segmentation framework (named 3DTU) for three-dimensional medical image segmentation tasks. This new framework processes images in an end-to-end manner and executes 3D computation on both the encoder side (which contains a 3D transformer) and the decoder side (which is based on a 3D DCNN). We tested our framework on two independent datasets that consist of 3D MRI and CT images. Experimental results clearly demonstrate that our method outperforms several state-of-the-art segmentation methods in various metrics.

## 1. Introduction

In the recent few years, deep convolutional neural networks (DCNNs) (Krizhevsky et al., 2012; Simonyan and Zisserman, 2014; He et al., 2016; Badrinarayanan et al., 2017; Huang et al., 2020; Pan et al., 2020) have achieved considerable progress in medical image segmentation (Long et al., 2015; Noh et al., 2015; Chen L.-C. et al., 2018; Tokunaga et al., 2019; Liu et al., 2022; Zhang et al., 2022). However, limited to the local receptive field of the convolutional filter, DCNN-based frameworks are incapable of capturing long-range dependencies from global features for semantic segmentation. To tackle this, several strategies can be considered. The first is to use the dilated convolution operation to enlarge the size of the receptive field of the convolutional filter (Yu and Koltun, 2015; Yang et al., 2017; Zhang et al., 2017; Liu et al., 2021). However, this enlarged local receptive field is still limited by the size of dilation. Another solution is to model the feature map as graph structures and investigate the long-range dependencies through the message passing mechanism of different graph learning models (e.g., graph convolution networks) (Li and Gupta, 2018; Chen et al., 2019; Li et al., 2020; Jia et al., 2021). Although these graph learning models have shown great potential in enhancing the global reasoning ability of DCNNs, they have very high requirements for computation and memory due to the constructed large-size graphs.

The attention mechanism (Hochreiter and Schmidhuber, 1997; Vaswani et al., 2017) is a computation scheme that tries to generate representations *via* different types of global features at each step. Since attention can be regarded as the conversion and transformation among the query (q), key (k), and value (v) triplet, attention computation is to generate the q based on the combination of the k–v pair. As it is natural to integrate a cycling computation in recurrent cells, traditional attention mechanisms are integrated within recurrent neural networks (e.g., Hochreiter and Schmidhuber, 1997; Cho et al., 2014), which inevitably impairs the efficiency of recurrent networks compared with linear/residual networks (Vaswani et al., 2017). To cope with this, Vaswani et al. (2017) proposed a transformer, a structure consisting of a series of identical encoder blocks connected with a series of identical decoder blocks, which all have no convolutional layers and are connected in a residual way. The original transformer supported by self-attention works exceptionally well in some tasks like machine translation but not in visual tasks (Chen et al., 2021). This is mainly due to the lack of convolution layers that makes the model struggle to detect local features.

For the aforementioned reasons, convolutional-based frameworks are still preferred for segmentation tasks. Although several other models (Goodfellow et al., 2014; Chen Y. et al., 2018) have been proven feasible, DCNNs remain to be one of the most effective methods. Multiple variants of DCNNs have been proposed to make the segmentation process more effective, one of the most crucial ones is the UNet (Ronneberger et al., 2015), which is a symmetric structure consisting of convolutional blocks with skip connections. These convolutional blocks have descending dimensions on the encoder side and ascending dimensions on the decoder side. However, due to the intrinsic fully convolution structure, UNet is suboptimal to relate local features to global representations with more variant distribution (Chen et al., 2021). To cope with the drawbacks of UNet, many methods have been proposed (Liu et al., 2018; Zhou et al., 2019; Diakogiannis et al., 2020; Huang et al., 2020). However, these methods are either very time-consuming or require heavy computations, which make it impossible to be applied to 3D objects.

Under such circumstances, the self-attention mechanism seems to be a nearly optimal solution. It is highly modulized and can stretch the number of self-attention cells according to the training environment. It can also train on vast datasets due to the training nature of attention. Therefore, researchers combined the transformer with convolutional layers for medical image segmentation (Li et al., 2022). On the one hand, the transformer encodes tokenized image patches from a CNN feature map as the input sequence for extracting global contexts. On the other hand, the decoder upsamples the encoded features, which are then combined with high-resolution CNN feature maps to enable precise localization.

However, this approach still has some obstacles, especially in the segmentation of 3D objects. This is partially due to transformers (Vaswani et al., 2017) requiring the input features to have temporal information. Since the self-attention does not compute with a clear direction, features have to be preprocessed with temporal info (e.g., cosine function) as input embeddings before training. Although this learning process can be seen as natural (scanning the features linearly and with order), it will restrict the performance of high-dimensional data. For example, many existing transformer approaches (Parmar et al., 2018; Huang et al., 2020; Chen et al., 2021) will cut the 3D object into 2D slice sequences to meet the temporal encoding requirement; however, the segmentation performance is actually worse because the 2D slice cutting will destroy the smoothness of the object in 3D space. Bi-directional transformer (Devlin et al., 2018) is a powerful upgrade version of transformer. It is a structure with no decoder and processes the inputs all at once with masks to create temporal/spatial continuity. However, we will show in the experiment section that bi-directional transformers can serve as a strong encoder but still struggles to get better results on 3D segmentation. To compensate for the loss of feature resolution brought by transformers, we propose 3D transformer UNet (3DTU), which employs a hybrid CNN–transformer architecture to leverage both detailed high-resolution spatial information from CNN features and the global context encoded by our new 3D bi-directional transformer module. We show that such a design allows our framework to preserve the advantages of self-attention mechanisms and also get considerably improved results on 3D image segmentation compared with previous U-Net-based or transformer-based methods. To sum up, our contributions to this article can be summarized as follows:

- We proposed a new 3D bi-directional framework to learn deep 3D features for medical image semantic segmentation.
- We designed a novel attention mechanism specifically suitable for network training and self-attention computation for 3D objects.
- We verified our new framework on multiple datasets, consisting of different imaging modalities (MRI and CT images) and different organs (placenta and lungs infected with COVID) and obtained state-of-the-art (SOTA) results. Our method beat baselines in performances on multiple metrics.

## 2. Related work

### 2.1. Fully convolutional network in medical image segmentation

Many studies have attempted to adopt convolutional networks to medical image segmentation. For example, Liu et al. (2018) presented a hybrid network consisting of both 3D CNN and 2D CNN in the brain image segmentation for Alzheimer's disease (AD) studies. Ronneberger et al. (2015) presented UNet, one of the most iconic encoder–decoder-based methods for medical image segmentation. Their method consists of convolutional blocks that have a U-shaped dimension variation. Specifically, from the input layer of the encoder to the input layer of the decoder, each block's dimension is descending. And the decoder has an ascending dimension that is matched to the encoder blocks. Such a design makes sure that the learning ability of the framework is powerful enough to find the abstract of the locality and output a global representation map. Several adjustments (e.g., Zhou et al., 2019; Huang et al., 2020) have been made to the original UNet model. For example, U-Net3+ (Huang et al., 2020) and its variations, although proved effective, still suffer from the locality-heavy learning scheme. Some researchers tried to boost the local reasoning of convolutional layers through the residual structure. For example, ResUNet (Diakogiannis et al., 2020) proposed a residual block between every two convolutional blocks on both the encoder side and decoder side as well as skip-connection between residual blocks with the same dimension between the encoder and decoder. Isensee et al. (2021) argued that the understanding of the datasets needed for training is more important than the network itself since most UNet-based moderations have achieved little progress. The authors proposed nnUNet, a robust network, that is designed based on the combination of 2D and 3D UNet. The authors also made different training configurations (normalization tricks, cropping, activation functions, etc.) based on the datasets.

### 2.2. Transformers

Transformers (Vaswani et al., 2017) were initially proposed for general NLP tasks and quickly gain widespread attention by beating previous most state-of-the-art results by a large margin. Devlin et al. (2018) converted the original transformer model into BERT, and introduced the called bi-directional transformers, which are proven effective again. Naturally, multiple efforts have been made to adjust the learning ability of transformers in the computer vision domain. Several variants of transformers have emerged recently. Parmar et al. (2018) presented one of the early works to adjust vanilla transformers by incorporating visual information. This model pre-processes each pixel of one image through a 1 × 1 convolution layer. Then, the embeddings are computed with positional embeddings before feeding into transformers for super-resolution tasks. In another attempt at visual tasks, Dosovitskiy et al. (2020) proposed Vision transformer (ViT), which presented a novel way of input embedding on visual information. It achieved state-of-the-art on ImageNet classification by directly applying transformers with global self-attention to full-sized images. Specifically, ViT flattens an image to fixed-sized pixels, which then be linearly added to positional embeddings before feeding to transformer encoders. Valanarasu et al. (2021) presented gated axial attention that creates a gated scheme to improve learning ability on the local scale.

### 2.3. Combination of UNet and transformer in medical image segmentation

Multiple attempts have been made to combine the UNet with transformer in both framework structure and inner encoder/decoder computation. TransUNet (Chen et al., 2021) consists of a series of transformer units as the encoder and the right half of the UNet as the decoder to generate predictions in medical image segmentation. Both the encoder and the decoder (Chen et al., 2021) are computed in a 2D scenario. Yun et al. (2021) introduced SpecTr, a framework that takes spectral normalization into the computation between convolution and attention blocks. Their methods achieved better results than the baseline when training on hyperspectral medical images. Wang et al. (2021) presented TransBTS that utilizes 3D CNN to extract input representations. UNet transformer, presented by Petit et al. (2021), replaces self-attention modules in transformer encoder/decoder cells by convolutional blocks and batch normalization computations. Another attempt is Swin-UNet (Cao et al., 2021), which instead replaces convolution blocks in the UNet-Structure network with self-attention modules. Several works follow similar methods including UNETR (Hatamizadeh et al., 2022b), SWIN UNETR (Hatamizadeh et al., 2022a), CoTr (Xie et al., 2021), nnFormer (Zhou et al., 2021), DS-TransUNet (Lin et al., 2022), UTNet (Gao et al., 2021), and PNS-Net (Ji et al., 2021). In UNETR, the authors presented a novel 3D transformer encoder and a voxel-wise loss for model training. For the positional embedding, they adopted a strategy from the Visual transformer, which divides the 3D images into 3D patches. The decoder in their work consists of several convolutional blocks in different dimensions and skip connections to the encoder. The SWIN UNETR is proposed for 3*D* multi-modal MRI brain image studies, which is different from the SWIN UNET that is proposed for 2D images. The CoTr utilized a DeTrans-encoder with a novel attention mechanism and a CNN-based decoder. The nnFormer utilizes CNN as part of an encoder, which leverages the ability of local feature extraction of CNN structures. Moreover, it utilizes transformer structures as its decoder and the second part of its encoder. There are two differences between our 3DTU and the nnFormer. First, we utilize a CNN-based structure (i.e., the right part of 3DUNet) as our decoder. Then, we design an attention mechanism that computes the attention scores from different directions.

The aforementioned methods adjust the transformers in visual tasks by introducing their own positional embedding rules. Although these rules are to an extent useful, their performance all suffers from the slicing of 3D data to adjust the positional embeddings. In this study, positional embeddings are not needed technically, even for 3D data. We modify the multi-head attention from its original form to a refined computation scheme that fully utilizes the potentials of transformer and UNet. More importantly, our encoder is a refined bi-directional transformer, which learns the feature from three (i.e., along x, y, and z) directions simultaneously.^1^

## 3. Methods

We propose a 3D UNet-based framework with bi-directional transformers (named 3DTU) in this work. The self-attention mechanism in the proposed bi-directional transformers can improve the ability of generalization of the framework encoder. We will delve into the technical details in this section.

**Figure 1:**
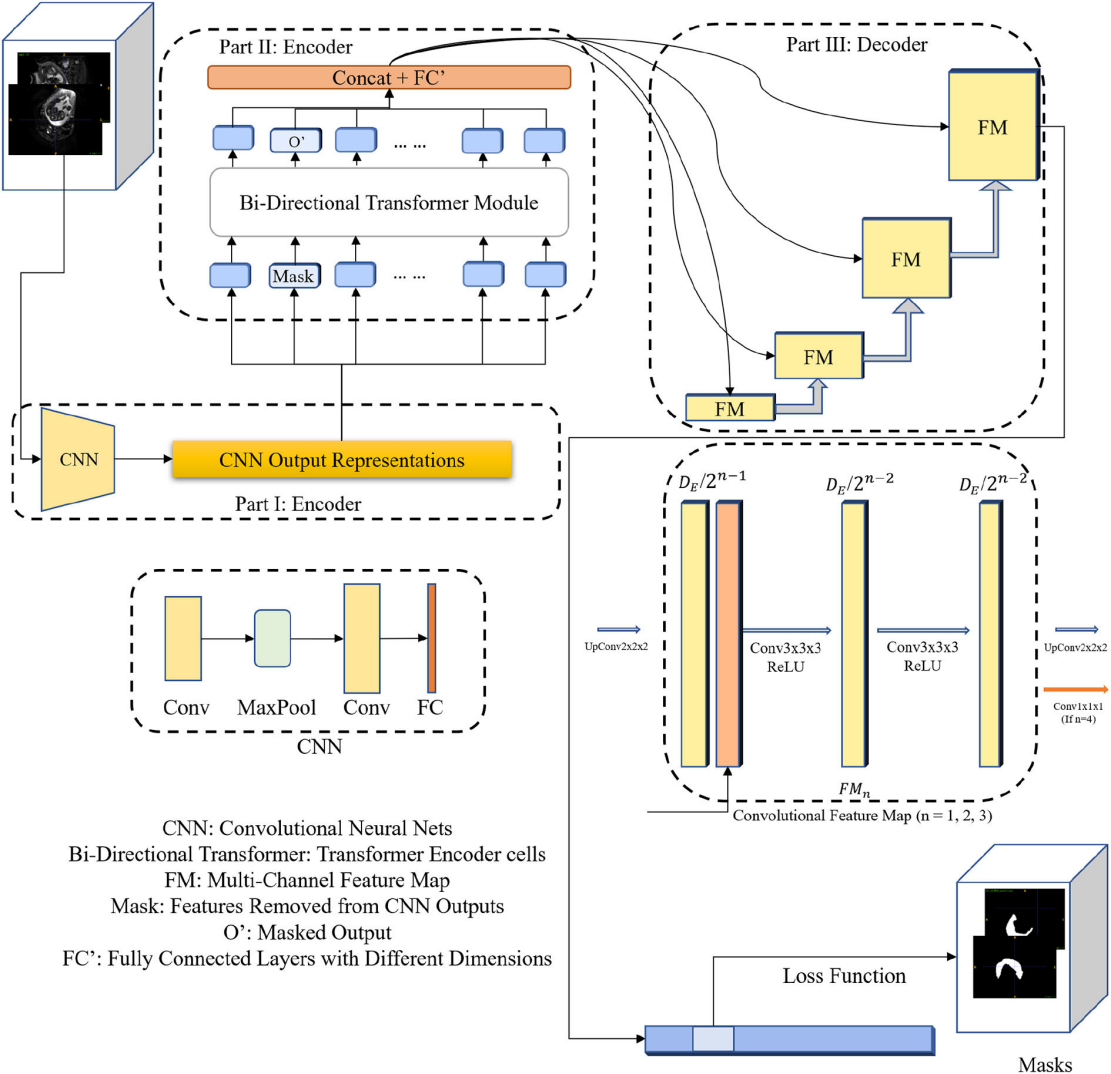
The diagram of the 3DTU framework in an encoder–decoder setting. The encoder consists of two parts including feature extraction and bi-directional transformer.

As shown in Figure 1, our proposed 3DTU is an encoder–decoder framework, where the encoder consists of two modules including a feature extraction module (see Part I in Figure 1) and a bi-directional transformer module (see Part II in Figure 1). Given a 3D image $I\in {ℛ}^{h\times w\times d\times c}$, where *h*, *w*, and *d* are the shapes of the image and *c* is the image channel number, the feature extraction module projects the 3D image *I* as a latent representation *X* *via* basic convolutional neural networks (CNNs). Then, the 3D bi-directional transformer cells take the latent representation *X* as input and yield the masked latent representation *X*_*M*_ by using Masked-LM (MLM) (Devlin et al., 2018) step by step. Finally, the decoder part utilizes the masked latent representations to reconstruct the segmentation predictions for loss computation.

### 3.1. Encoder with 3D bi-directional transformer

As aforementioned, the encoder of the 3DTU consists of two parts. The first part of the encoder is a CNN-based feature extraction module. We aim to convert the original 3D image (*I*) into an iso-dimensional latent cube representation ($X\in {ℛ}^{1\times p\times p\times p}$) *via* this module as assistance to capture the image locality for transformer modules, since the transformer module may not have enough ability to capture the image local features. We will show this point in the ablation studies. Particularly, the feature extraction module includes two convolutional layers followed by a fully-connected (FC) layer and a max-pooling layer in between the two convolutional layers. The FC layer is used to adapt the feature dimension.

**Figure 2:**
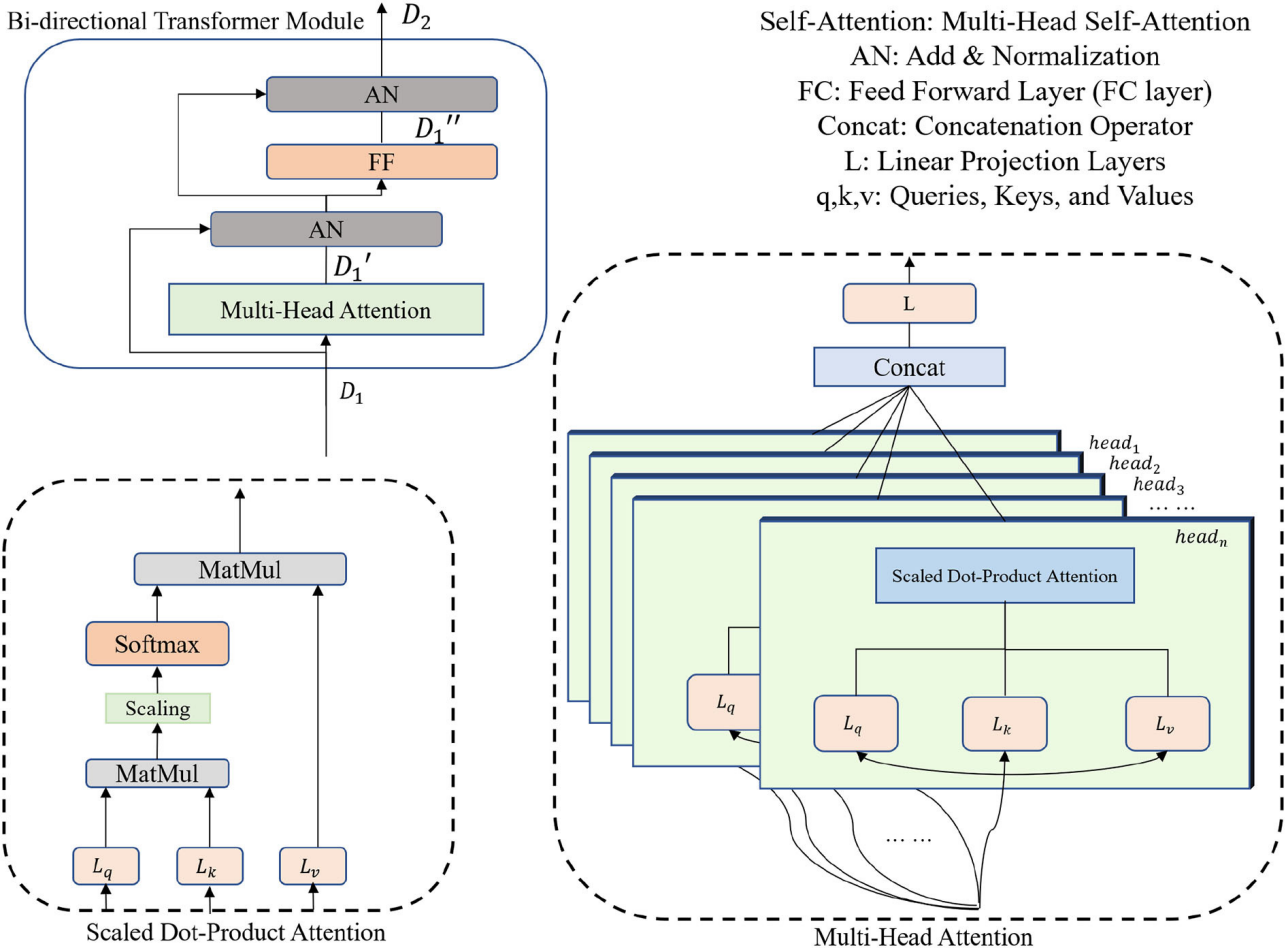
Encoder Part II: bi-directional transformer with a multi-head attention mechanism.

The bi-directional transformer module takes the latent cube representation *X* as input and computes multi-head attentions with the MLM strategy (Devlin et al., 2018). Details of the bi-directional transformer module are shown in Figure 2. In general, each cell in the bi-directional transformer module generates the latent feature map *X*_1_ by the following steps:


(1) $$\begin{array}{l} \begin{array}{lll} X^{{}^{\prime }} & = & Att(Norm(X))+X,\\ X^{{}^{\prime \prime }} & = & FF(Norm(X^{{}^{\prime }})), \end{array}\\ \begin{array}{lll} X_{1} & = & X^{{}^{\prime }}+X^{{}^{\prime \prime }}, \end{array} \end{array}$$

where *Att*(·) is the multi-head self-attention operation, *Norm*(·) is a 3D normalization operation, and *FF*(·) is the feed forward layer (i.e., FC layer). + denotes a pixel-wise add operation. Particularly, the multi-head attention is computed as follows:


(2) $$\begin{array}{l} \;Att\_head_{i}^{x,y,z}=SDP(Q,K,V)\times W,\\ MultiHead(Q,K,V)=Concat(head_{i}^{x},head_{i}^{y},head_{i}^{z}), \end{array}$$

where *SDP*(·) is the Scaled Dot-Product Attention, *W* is the trainable parameters for linear projections (i.e., *L*_*q*_, *L*_*k*_, and *L*_*v*_ in Figure 2) and *Concat*(·) denotes a concatenation operation. *Q, K*, and *V* are the query-key-value triplets defined by the transformer cell. Note that our proposed attention mechanism can yield the attention score by scanning the query-key-value triplets in three different directions (i.e., along x, y, and z axes, respectively), which gain plentiful discriminative and anisotropic semantic information for the 3D image segmentation.

### 3.2. UNet-based decoder

As shown in Figure 1, we utilize convolutional blocks with ascensional dimensions in the decoder part. A residual connection is adopted between the encoder side and the decoder side. Particularly, a cascaded of multi-channel feature map (FM) blocks are integrated into the decoder part, each of which contains two 3 × 3 × 3 convolutional layers and an upsampling layer. The channel number of feature maps reduces by half after each FM block. In the last FM block, instead of upsampling layer, a 1 × 1 × 1 convolutional layer is used to generate final segmentation predictions.

### 3.3. Loss function and supervision manner

Since the MLM strategy is used in the encoder part, where a portion of image features are masked (i.e., set to 0 values) and the other portions remain the same. Hence, our goal is to use the uncovered portions to predict the masked portions (Devlin et al., 2018), in which the loss is only estimated based on the masked regions. Particularly, the loss function can be formulated as:


(3) $$\begin{array}{l} ℒ=\alpha \times \ell_{dice}\left({ŷ}_{mask},y_{mask}\right)+\left(1-\alpha \right)\times \ell_{BCE}\left({ŷ}_{mask},y_{mask}\right), \end{array}$$

where ŷ_*mask*_ and *y*_*mask*_ are the masked regions of segmentation prediction and ground truth, respectively. α ∈ [0, 1] is the loss weight.

## 4. Experiments

### 4.1. Datasets

We used three datasets obtained from different modalities for this study, including Placenta MRI (Placenta) dataset, COVID-19 CT lung and infection segmentation (Covid20) dataset, and Multi-Atlas Labeling Beyond the Cranial Vault (Synapse) dataset. Details of data description and preprocessing are shown below.

Placenta MRI dataset was collected from the Washington University in Saint Louis (WUSTL) (Sun et al., 2022), where all data were de-identified before processing. The data collection and related studies were approved by the Institutional Review Board at the WUSTL. A total of 81 MRI scans were collected from 46 pregnant patients (mean age = 23.91 ± 3.02 yo, mean BMI = 25 ± 3.66 at recruitment) with normal singleton pregnancy who underwent MRI during the third trimester, by a Siemens 3T VIDA scanner. Of the 46 patients, 21 patients had the single scan and 25 patients had multiple longitudinal scans. The average gestational ages (GA) during MRI scans were 34.12 ± 1.07 weeks (Min GA 28 weeks 3 days, max GA 38 weeks 6 days). T2-weighted MRI of the entire uterus was acquired with a 2D EPI sequence in the left lateral position. The MRI data has a fixed acquisition matrix of 128 × 128 × 115, and variable voxel sizes from 3 × 3 × 3 mm to 3.5 × 3.5 × 3.5 mm, up to the patient's size. Manual segmentation of the placenta regions was conducted by experienced radiologists for all MRI images.

COVID19-CT-Seg20 dataset (Covid20) contains 20 COVID-19 3D CT images, where lungs and infections were annotated by two radiologists and verified by an experienced radiologist^2^ (Jun et al., 2021). We only focused on the segmentation of the COVID-19 infections in this study, since it is more challenging and important.

Multi-atlas labeling beyond the cranial vault (Synapse) dataset.^3^ We use the 30 abdominal CT scans from the MICCAI 2015 Multi-Atlas Abdomen Labeling Challenge. These scans were captured during the portal venous contrast phase with variable volume sizes (512 × 512 × 85–512 × 512 × 198) and field of views (approximately 280 × 280 × 280 *mm*^3^–500 × 500 × 650 *mm*^3^). The in-plane resolution varies from 0.54 × 0.54 *mm*^2^ to 0.98 × 0.98 *mm*^2^, while the slice thickness ranges from 2.5 to 5.0 *mm*. We report the average experimental results on eight abdominal organs (aorta, gallbladder, spleen, left kidney, right kidney, liver, pancreas, spleen, and stomach) with 5-fold validation.

### 4.2. Implementation details

In the pre-processing step, we simply normalized the intensities of each 3D image to zero mean and unit variance. In the training phase, we applied data augmentation techniques to reduce potential overfitting, including random rotation of the image by 90° along three dimensions and adjusting the brightness of the top 3% pixels. The training iterations were set to 10^5^. We trained the model using the Adam optimizer with a batch size of 1 and synchronized batch normalization. The initial learning rate was set to 1*e*−2 and was decayed by ${\left(1-\frac{current\text{\_}epoch}{max\text{\_}epoch}\right)}^{0.9}$. We also regularized the training with dropout in the transformer cells. All experiments are conducted using a 5-fold cross-validation, based on Pytorch 1.7.1 on a workstation with 2 NVIDIA TITAN RTX GPUs. The data division on the public Covid20 dataset is adopted by following the division strategy given by Qiu et al. (2021).

As aforementioned, our encoder consists of two parts. In the feature extraction module, we used a CNN network with two convolutional layers, one max-pooling layer, and one 1-D fully-connected layer with the direction of *x*−*y* plane to *z* coordinate to convert the representations with the original dimension to a cube. The first convolutional layer, with a kernel size of 3 × 3 × 3, embeds the input 3-D image into local representation maps, while the second convolutional layer project the local representation maps for the second part of the encoder *via* a linear transformation. The output dimension of the feature extraction module is converted (i.e., reshape) to $X\in {ℛ}^{1\times 256\times 256\times 256}$. In the bi-directional transformer module, we utilize multiple transformer cells with the bi-directional self-attention mechanism. Specifically, the input embedding strategy that we adopted is Masked LM (MLM) (Devlin et al., 2018). The Masked LM has been proven to be useful within the previous BERT paper (Vaswani et al., 2017), where the image portion masked in the encoder is matched to that in the loss computation stage. Moreover, since we do not embed the data with the positional encoding in our framework, we require a way to learn the 3D representations through a certain sequence. MLM can well meet this requirement. We set the number of transformer cells as 12, 6, and 6 for Placenta, Covid20, and Synapse datasets, respectively. The number of heads within each transformer cell is 15, where each direction (i.e., *x*−*y*, *x*−*z*, and *y*−*z* plane) contains five heads to compute self-attention scores. The length of each mask is set to 16, 32, and 32 for the Placenta, Covid20, and Synapse datasets, respectively. Each cube representation is divided into 16 parts in the training phase.

### 4.3. Baseline settings and evaluation metrics

To evaluate our 3DTU's performance, we choose the following frameworks as baselines: 2DU-Net (Ronneberger et al., 2015), 3D U-Net (Çiçek et al., 2016), UNet++ (Zhou et al., 2019), TransUNet (Chen et al., 2021), ViT (visual transformer) (Dosovitskiy et al., 2020), nnFormer (Zhou et al., 2021), and nnUNet (Isensee et al., 2021). Both 2D and 3D UNet are FCN-based encoder–decoder structures with convolutional blocks and skip-connections between the encoder and decoder. The UNet++ is a nested-connected encoder–decoder structure, where each convolutional block is connected to all other blocks. The TransUNet is an encoder–decoder network, where the encoder of UNet is replaced by a 2D transformer including a positional embedding scheme followed by a visual transformer (ViT). The nnFormer is a 3D UNet-type framework that replaces the convolutional blocks with three different novel attention mechanisms.

The metrics we used to evaluate our 3DTU include mIoU, DICE score, and Hausdorff Distance (HD). IoU is the area of overlap between the predicted segmentation and the ground truth divided by the area of union between them. For binary (two classes) or multi-class segmentation, the mean IoU (mIoU) of the image is calculated by taking the IoU of each class and averaging them. DICE score is the harmonic mean of precision and recall of the segmentation results. mIOU and DICE scores are two overlap-based metrics measuring the similarity between the ground truths and segmentation predictions. The range of mIOU and DICE scores is from 0 to 1 and the larger value indicates better segmentation performance. The directed average Hausdorff distance (HD) from point set X to Y is computed by the sum of all minimum distances from all points from point set X to Y divided by the number of points in X. HD is a shape distance-based metric, which measures the dissimilarity between the surfaces of the segmentation results and the related ground truths. A lower value of HD indicates better performance.

### 4.4. Comparative experiments

**Figure 3:**
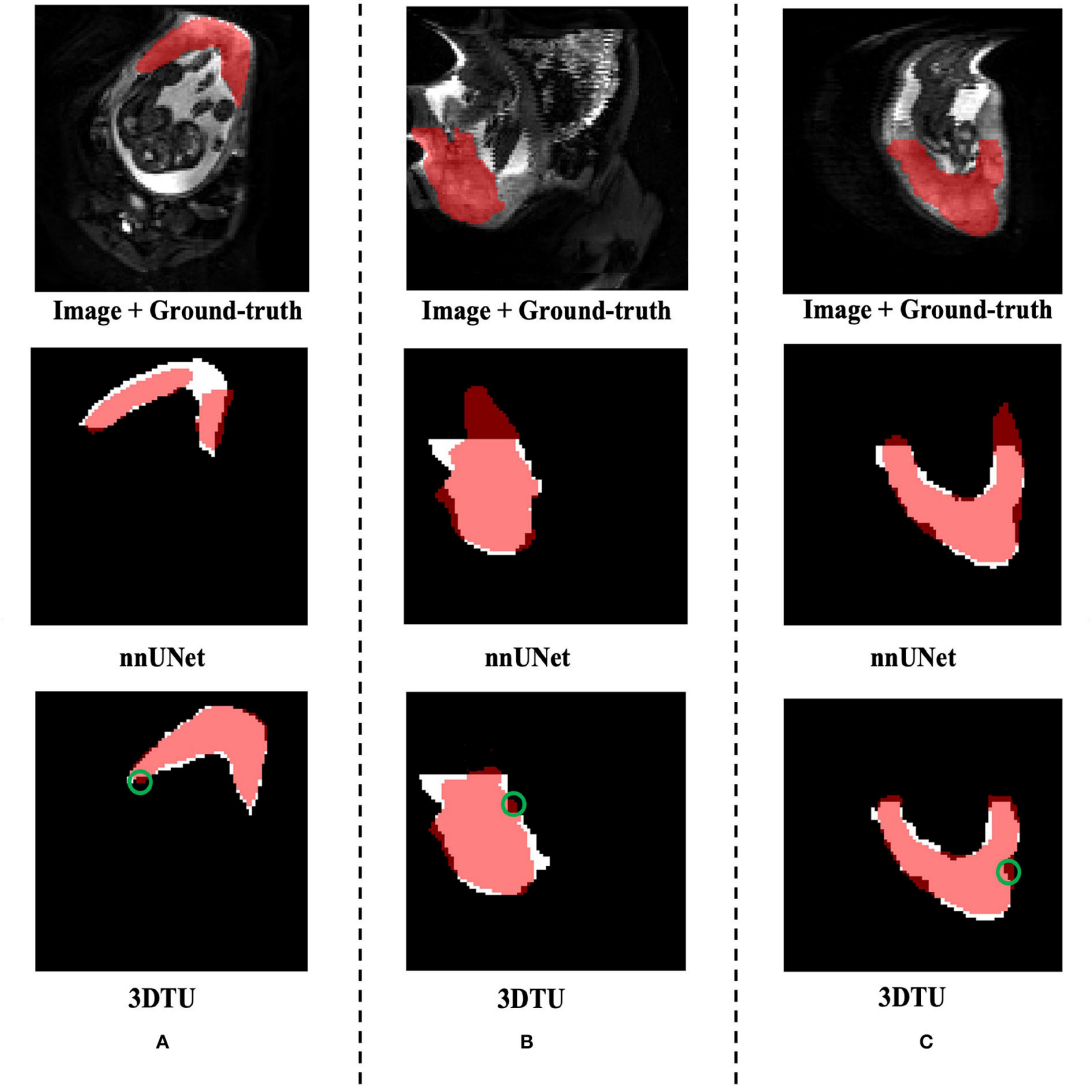
Visualization of the segmentation results on the Placenta dataset produced by our 3DTU and nnUNet. Columns **(A–C)** show the *x*–*y* plane, *y*–*z* plane, and *x*–*z* plane of 3D segmentation predictions, respectively. The true-positive regions are highlighted in pink. The false-negative regions are highlighted in red (e.g., the green circle regions in the last row). Better view with colors and zooming in.

**Figure 4:**
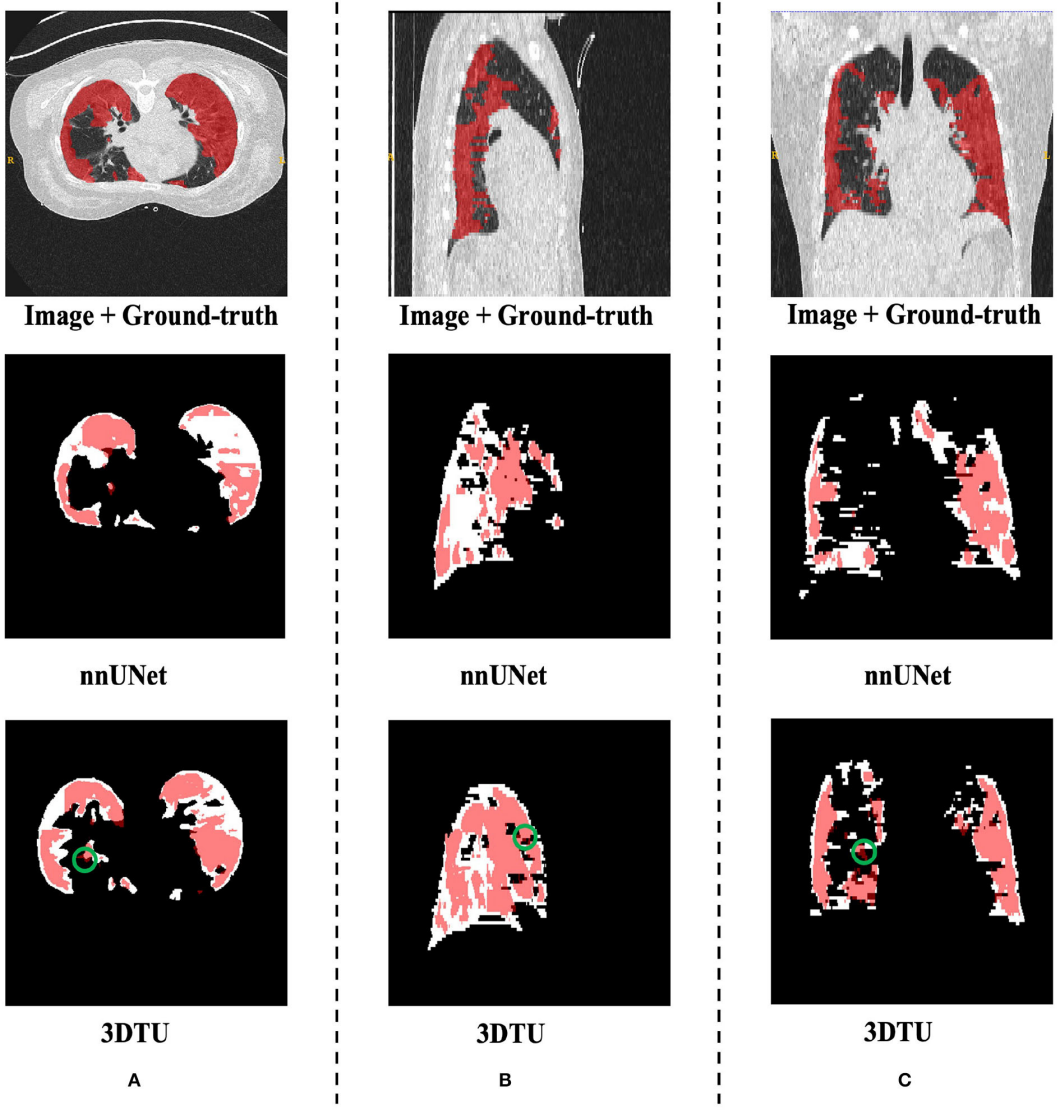
Visualization of the infection segmentation results on the Covid20 dataset produced by our 3DTU and nnUNet. Columns **(A–C)** show the *x*–*y* plane, *y*–*z* plane, and *x*–*z* plane of 3D segmentation predictions, respectively. The true-positive regions are highlighted in pink. The false-negative regions are highlighted in red (e.g., the green circle regions in the last row). Better view with colors and zooming in.

**Figure 5:**
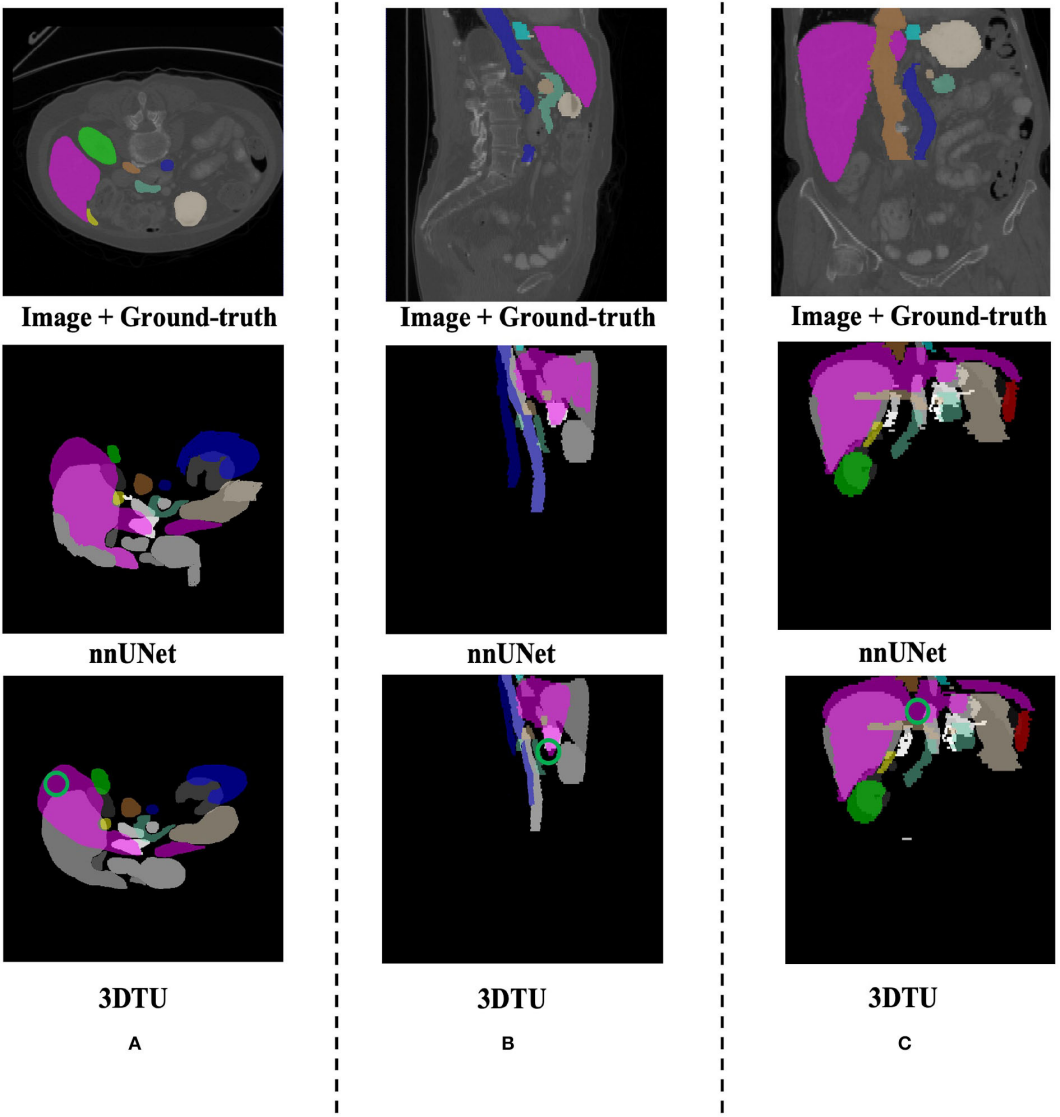
Visualization of the segmentation results on the Synapse dataset produced by our 3DTU and nnUNet. Columns **(A–C)** show the *x*–*y* plane, *y*–*z* plane, and *x*–*z* plane of 3D segmentation predictions, respectively. The green circle indicates part of false-negative regions. Better view with colors and zooming in.

Table 1 provides the performance of our proposed 3DTU and the six competing baselines, including 2D UNet (Ronneberger et al., 2015), 3D UNet (Ronneberger et al., 2015), UNet++ (Zhou et al., 2019), TransUNet (Chen et al., 2021), visual transformer (ViT) (Dosovitskiy et al., 2020), and nnFormer (Zhou et al., 2021) on the Placenta and Covid20 datasets. It shows that our 3DTU outperforms all competing baseline methods consistently in terms of mIOU and DICE scores on both datasets, while beating most of the methods in the baseline in the Synapse dataset, indicating that the segmentation results of our models match well with the ground truth. For example, our proposed 3DTU outperforms baselines with at least 0.48% and 0.44% increases in DICE scores on Placenta and Covid20 datasets, respectively. This may attribute to the attention mechanism proposed in the 3DTU, which can compute the attention scores from three different directions to yield discriminative and anisotropic semantic features for 3D images. In general, the transformer-based methods (e.g., TransUNet, ViT, etc.) perform better than the other baseline methods. In addition, we visualized the segmentation results of our 3DTU and the best baseline method (i.e., nnUNet) on three datasets in Figures 3–5, respectively.

**Table 1:** Quantitative segmentation results of different methods on two datasets, where mIOU and DICE are in %.

	**Placenta dataset**			**Covid20 dataset**			**Synapse dataset**		
	**mIOU**	**DICE**	**HD95**	**mIOU**	**DICE**	**HD95**	**mIOU**	**DICE**	**HD95**
2D UNet	67.6	72.3	12.0	73.6	78.3	112.5	56.3	60.6	45.7
3D UNet	72.5	78.6	10.7	78.1	84.0	97.6	59.4	62.2	42.2
UNet++	74.5	77.1	8.2	80.3	84.6	63.0	67.1	73.7	34.0
TransUNet	73.6	80.0	7.4	83.1	89.2	45.8	70.2	77.5	31.7
ViT	72.9	79.7	8.5	84.2	89.0	70.3	65.3	67.9	36.1
nnFormer	78.3	82.1	10.2	81.0	89.9	66.2	81.8	86.6	10.6
nnUNet	78.9	83.6	8.7	90.3	91.6	59.9	84.2	89.8	16.6
3DTU (Ours)	79.8	84.0	7.2	90.5	92.0	59.4	85.0	87.3	18.4

The best results are shown in red and the second-best results are shown in blue.

### 4.5. Ablation study

We conducted an ablation study on both datasets (i.e., Placenta and Covid20) to evaluate the effectiveness of each part in our 3DTU framework. Our 3DTU is an encoder–decoder-based framework, where the encoder consists of a CNN networks part as well as a bi-directional transformer (BiT) part, where the decoder is in the UNet decoder setting. Hence, we designed the following four experiments in our ablation study.

- We removed the CNN networks in the encoder and directly fed the input images to the BiT part.
- We removed the BiT part in the encoder and directly connected the CNN networks to the UNet decoder.
- We removed the UNet decoder part and considered the BiT as both (part of) encoder and decoder.^4^
- We designed a comparative experiment where we trained 3DTU with positional encoded representations. We encoded the representations at the input of the transformer encoder.

The results in Table 2 show the effectiveness and necessity of all the sub-parts in our 3DTU. The results in Table 3 indicate that positional encoding is not necessary in our framework since our attention mechanism can process the 3D data as a whole. Compared with the 3DTU w/o positional encoding, the segmentation dice scores yielded by 3DTU with positional encoding are not changed or even decreased. When we removed the CNN networks and only utilized BiT as the encoder (see results of BiT+Unet decoder in Table 2), the segmentation performance decreased on both datasets (e.g., DICE decrease from 84.0 to 66.9% and from 92.0 to 72.8% on Placenta and COVID datasets, respectively). This indicates an essential role of CNN-based convolutional layers in the encoder, without which the self-attention transformer layers may not localize the raw image pixels precisely. Meanwhile, the segmentation performance increase when we use BiT instead of UNet as a decoder (see results of CNN + UNet Decoder and CNN + BiT). This manifests that, compared with UNet-based methods, the (bi-directional) transformers are more powerful in boosting the segmentation results.

**Table 2:** Dice scores (in %) of our 3DTU on three datasets.

**DICE score**	**Placenta dataset**	**Covid20 dataset**	**Synapse dataset**
CNN + UNet decoder	68.6	74.3	59.5
BiT + UNet decoder	66.9	72.8	70.2
CNN + BiT	80.0	89.2	65.1
3DTU	**84.0**	**92.0**	**87.3**

The best results are shown in bold.

**Table 3:** Dice scores (in %) of our 3DTU running on data that has been preprocessed with/without positional encoding.

	**Placenta dataset**	**Covid20 dataset**	**Synapse dataset**
3DTU w/o Positional encoding	84.0	92.0	87.3
3DTU with Positional encoding	82.7	92.1	86.8

### 4.6. Parameter analysis

**Figure 6:**
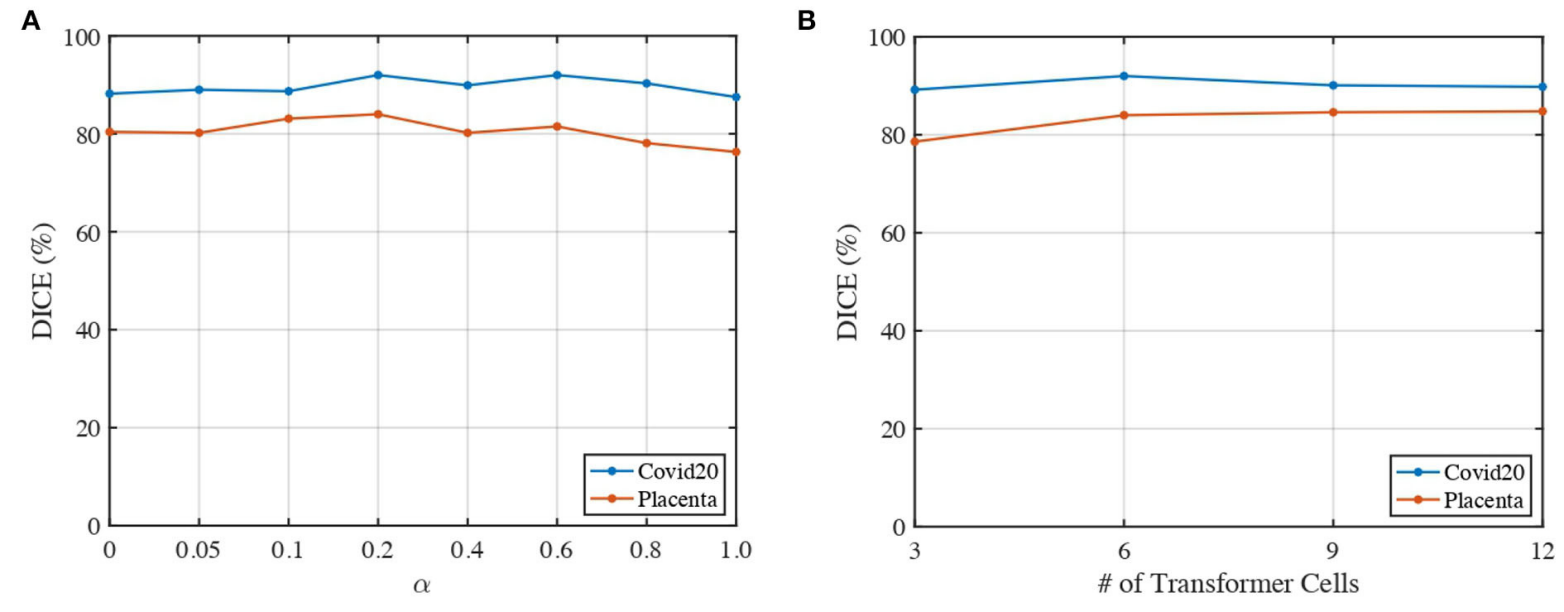
Impacts of α and number of transformer cells on segmentation performance. **(A)** Dice of 3DTU vs. α. **(B)** Dice of 3DTU vs. number of transformer cells.

We analyze the impact of two parameters, including the loss weights α and the number of transformer cells, on the segmentation performance of our proposed 3DTU across two datasets in Figure 6. In general, Figure 6 indicates that the segmentation results performed by our 3DTU are consistent. Figure 6A shows that the dice results increase and then decrease with the increase of α from 0 to 1. The best dice scores are achieved when α = 0.2 on both Placenta and Covid20 datasets. Figure 6B shows that the segmentation performance improves when increasing the number of transformer cells from 3 to 6. However, the performance will keep stable (on the Placenta dataset) or even slightly decrease (on the Covid20 dataset) when the framework goes deeper. The reason for the slight decrease in the performance of the Covid20 dataset may result from the small size of the dataset. Only 20 3D images are included in the Covid20 dataset, which may not facilitate the training process when the network goes deep. Moreover, our 3DTU has a total of 70M parameters (when training on the Covid20 dataset and the Synapse dataset), which is more than 2D UNet (7M) and 3D UNet (17M) but beats the other transformer-based or hybrid framework in the baseline (the TransUNet has 80M parameters, and nnFormer has 158M parameters).

## 5. Conclusion

In this article, we propose a novel 3D transformer UNet (3DTU) framework to capture global contextual information for 3D medical image segmentation. A new attention mechanism is proposed with our 3DTU framework, which is especially suitable for computing self-attentions for 3D objects. The experimental results on two 3D medical image datasets demonstrate that our method can outperform several state-of-the-art segmentation baselines. In the future, we plan to explore how to reduce the computation loads in transformer layers, which may improve the efficiency of most current transformer-based methods.

## Data availability statement

The Covid20 dataset is from the community of Coronavirus Disease Research-COVID-19 (Jun et al., 2021) and is available *via* https://zenodo.org/record/3757476#.Y1NGmy1h1B1. The Placenta dataset is available upon request.

## Author contributions

XF took charge of conception, design, method implementation, statistical analysis, and manuscript writing. ZS and YW took charge of data collection and preprocessing. ZS, EZ, HH, and YW took charge of experimental design, results discussion, and manuscript proofreading. HT and LZ took charge of project design, analysis, interpretation, and manuscript writing/revising. All authors contributed to the article and approved the submitted version.

## Footnotes

^1^We use the term “bi-directional” by following previous studies. However, our 3DTU learns the features from three directions instead.

^2^ https://zenodo.org/record/3757476#.Y1NGmy1h1B1

^3^ https://www.synapse.org#!Synapse:syn3193805/wiki/217789

^4^It shows in Devlin et al. (2018) that the bi-directional transformer can serve as both encoder and decoder.
